# IoT-Based Geotechnical Monitoring of Unstable Slopes for Landslide Early Warning in the Darjeeling Himalayas

**DOI:** 10.3390/s20092611

**Published:** 2020-05-03

**Authors:** Minu Treesa Abraham, Neelima Satyam, Biswajeet Pradhan, Abdullah M. Alamri

**Affiliations:** 1 Discipline of Civil Engineering, Indian Institute of Technology Indore, Madhya Pradesh 453552, India; minu.abraham@iiti.ac.in (M.T.A.); neelima.satyam@iiti.ac.in (N.S.); 2 Centre for Advanced Modelling and Geospatial Information Systems (CAMGIS), Faculty of Engineering and Information Technology, University of Technology Sydney, Sydney P.O. Box 123, Australia; 3 Department of Energy and Mineral Resources Engineering, Sejong University, Choongmu-gwan, 209 Neungdong-ro, Gwangjin-gu, Seoul 05006, Korea; 4 Department of Geology & Geophysics, College of Science, King Saud University, P.O. Box 2455, Riyadh 11451, Saudi Arabia; amsamri@ksu.edu.sa

**Keywords:** landslides, early warning system, IoT, monitoring, sensors, Indian Himalayas

## Abstract

In hilly areas across the world, landslides have been an increasing menace, causing loss of lives and properties. The damages instigated by landslides in the recent past call for attention from authorities for disaster risk reduction measures. Development of an effective landslide early warning system (LEWS) is an important risk reduction approach by which the authorities and public in general can be presaged about future landslide events. The Indian Himalayas are among the most landslide-prone areas in the world, and attempts have been made to determine the rainfall thresholds for possible occurrence of landslides in the region. The established thresholds proved to be effective in predicting most of the landslide events and the major drawback observed is the increased number of false alarms. For an LEWS to be successfully operational, it is obligatory to reduce the number of false alarms using physical monitoring. Therefore, to improve the efficiency of the LEWS and to make the thresholds serviceable, the slopes are monitored using a sensor network. In this study, micro-electro-mechanical systems (MEMS)-based tilt sensors and volumetric water content sensors were used to monitor the active slopes in Chibo, in the Darjeeling Himalayas. The Internet of Things (IoT)-based network uses wireless modules for communication between individual sensors to the data logger and from the data logger to an internet database. The slopes are on the banks of mountain rivulets (jhoras) known as the sinking zones of Kalimpong. The locality is highly affected by surface displacements in the monsoon season due to incessant rains and improper drainage. Real-time field monitoring for the study area is being conducted for the first time to evaluate the applicability of tilt sensors in the region. The sensors are embedded within the soil to measure the tilting angles and moisture content at shallow depths. The slopes were monitored continuously during three monsoon seasons (2017–2019), and the data from the sensors were compared with the field observations and rainfall data for the evaluation. The relationship between change in tilt rate, volumetric water content, and rainfall are explored in the study, and the records prove the significance of considering long-term rainfall conditions rather than immediate rainfall events in developing rainfall thresholds for the region.

## 1. Introduction

Landslide hazards result in fatalities in urban areas, agricultural lands, and road corridors on slopes. In a global database of non-seismic landslides, it was inferred that 75% of the landslides that occurred between 2004 to 2016 took place in Asia, with a major share of events in the Himalayan Arc [1]. Rainfall is the primary triggering factor, so the relationship between rainfall and landslides in the Indian Himalayas have been explored in detail [2,3,4,5,6,7,8]. These thresholds can be used to determine the temporal probability of the occurrence of landslides as a part of a landslide early warning system (LEWS). The thresholds can be estimated using empirical methods [9,10,11,12], probabilistic methods [8,13,14], and/or physical methods [15,16], and we can use other tools like geographic information system (GIS) [17] and global positioning system (GPS) [18,19] and new technologies in order to make the calculation processes automatic [20,21]. When long-term monitoring data is not available for the study area, it is often difficult to identify the triggering rainfall corresponding to landslides [22]. A major drawback observed in many of these methods is the high number of false alarms. The thresholds are based on the assumption that the in-situ conditions remain unchanged with respect to time and, hence, historical data can be used to predict future events. This does not always hold true, so LEWS should consider the effect of real-time site conditions. When thresholds are developed on a regional scale, the effect of the local site conditions and material heterogeneity is neglected [23]. In addition, the empirical thresholds are statistically based and do not take the complex hydrological processes and failure mechanisms into account when deriving the thresholds. Therefore, for accurate prediction of landslide hazards, a field monitoring system is critical to understand the in-situ conditions. It is an extensively accepted fact that slope failures are often preceded by cracks due to slope deformations. If the minor movements of the slopes can be monitored effectively, this can be correlated with the potential occurrence of displacement [23] in the locale. The authorities can make a better judgment about the warning that should be given to the public based on real-time monitoring.

Several techniques are being used for field monitoring of unstable slopes. The most common method is scheduled inspection at regular time intervals. Abnormal slope deformations along transportation corridors are identified as potential gravity-induced slope failures. Rainfall-induced landslides are much faster than those induced by gravity, and this approach is not effective in forecasting such events. Satellite based and ground based networks like synthetic aperture radars (SAR), light detection and ranging (LiDAR), etc. are also used to monitor the slopes in real time [24,25,26,27,28,29]. Multi-interfero-metric techniques like Permanent Scatterer Synthetic Aperture Radar Interferometry (PSInSAR) can be used for ground displacement studies with very high accuracy [30,31]. PSInSAR identifies radar targets with high phase stability throughout the duration of observation. The targets of PSInSAR are point-wise objects available in urban areas. The geophysical parameters are extracted from deterministic points. To overcome this limitation, an advanced algorithm called SqueeSAR is now used [32]. SqueeSAR extracts the parameters from point-wise objects and distributed scatterers and processes them jointly [32]. If better-resolution images can be obtained at a lesser expense, this is a promising approach in predicting slope failures. In-situ ground-based observations are another emerging method for the real-time monitoring of slopes. With the advances in wireless networks integrated with the Internet of Things (IoT), data from anywhere can be accessed in real time using machine to machine communication. Different types of mechanical and electrical systems are used to predict the failure for early warning. Extensometers are used for this purpose to find out the distance between the moving soil mass from the stable one [33]. The challenge is to have knowledge about the part of slope that has a probability to move. Inclinometers can also be used for deep-seated installations [34] but cannot be chosen for low-cost installation in large areas due to the expense and expertise required for installation. Usage of several wireless sensors are reported in the literature for monitoring landslides [35,36,37,38,39,40,41], and in this study, a more reliable approach is attempted by using tilt sensors [38,42], which are found to be an economically viable solution.

The technique was first developed and tested by monitoring several real slopes in Japan [38,43]. The sensors are found to be effective in monitoring shallow landslides. Similar sensors were installed in China [44], and by integrating in-situ monitoring and hydro-mechanical analyses, an early warning system was proposed to forecast landslides in the Wenchuan region. In the Indian context, using tilt meters is a cost-effective alternative to the traditional extensometer approach. The hydrogeological settings of the terrain are completely different from the locations in China and Japan where the sensors have been already used. Before incorporating the sensor network as a part of a LEWS, it is crucial to evaluate its performance, particularly for the given study area, in correlation with the field observations. Also, the mechanism of failure in the region needs to be studied in detail to understand the failure pattern to derive a regional specific LEWS. The study region is experiencing continuous subsidence, and the failure occurs in a very slow rate in the uphill zone. Hence, sensitive monitoring systems are required to find the tilt angles and predict a possible failure well before the actual occurrence. This study aims at understanding the applicability of tilt sensors for slope monitoring in the study area, and it is the first of its kind for Kalimpong using real-time monitoring. The use of la ow-cost sensor network will make it possible to install more sensors for effective monitoring of slopes and can aid in the development of the LEWS for the region. The recorded tilt angles from the sensors are compared with the rainfall conditions and moisture content data in order to understand the relationship between rainfall and displacements, thereby evaluating the effect of daily rainfall and antecedent rainfall on tilt angles and volumetric moisture content.

## 2. Study Area

**Figure 1:**
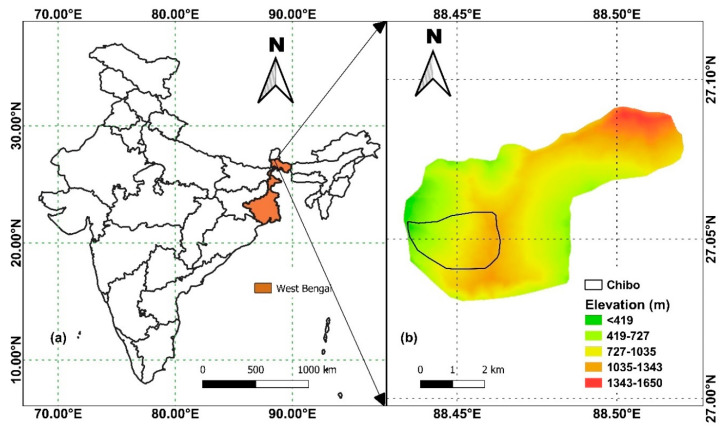
Location of the study area (**a**) in India, and (**b**) digital elevation model of Kalimpong.

The study was conducted in a sinking zone of the Darjeeling Himalayas named Chibo. The area is located within the Kalimpong town of West Bengal, India (Figure 1). It belongs to the Teesta basin, which is along the western slope of Kalimpong. During the monsoon season, displacements are observed in the region due to high precipitation and drainage density. The area is drained by several streams and their tributaries, known as jhoras in the local dialect. These jhoras are untrained, and the surface runoff during monsoon makes the banks highly unstable. The sub-parallel to the dendritic drainage pattern indicates strong structural control [24]. The study area is surrounded by valleys and dissected hills, and this part of the Himalayas involves the Fold-Thrust-Belt (FTB) and belongs to Zone IV of India’s seismic zonation map. Geologically, Chibo consists of phyllite, schist, quartzite, and sheared granite gneiss [5]. Most of the area is covered with thick overburden materials with intermittent exposures of weathered rocks. The overburden materials are of varying particle sizes and debris type. A major share of the soil includes silt, sand, and gravel. Sandy soils are found in the eastern part of the Teesta basin [25]. With an increase in elevation, the particles change from gravels to rocks. Rapid urbanization of the region has increased the fatalities associated with landslides. Recent land use modifications associated with urbanization have increased geological instability, which has led to loss of lives and assets. The complex land movements in Kalimpong includes surface erosion, subsidence, shallow landslides, and debris flows. Unlike the previous studies using MEMS sensors [44,45], where only shallow landslides were monitored, very slow displacements are observed in the study region due to toe erosion and the subsequent settlement of hilly terrains.

**Figure 2:**
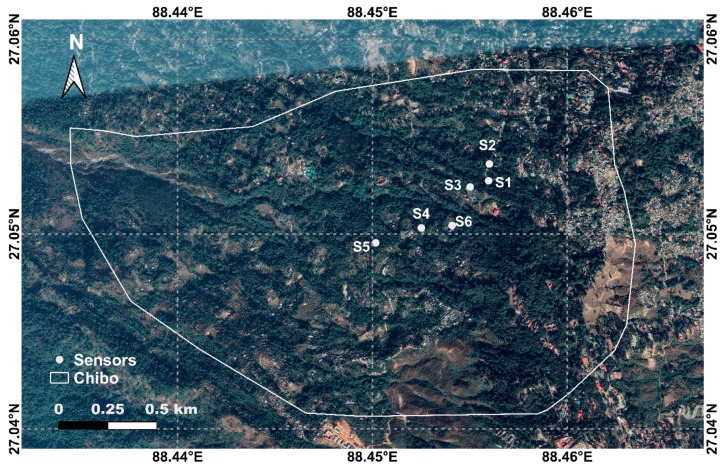
Location of tilt sensors installed in Chibo.

The entire region is suffering from erosion and scouring along the jhoras. The erosion downhill is in turn resulting in subsidence of the upper hilly portions as well. The construction activities also affected the drainage network, and surface runoff is allowed to enter the thick overburden debris from past slides. The most affected zones were selected for field monitoring using tilt sensors. The locations (Figure 2) were identified after field surveys conducted in 2016 [46]. Two mountain rivulets (OC jhora and Pyarieni jhora) were identified as critical after detailed field inspections and discussions with the local communities.

**Figure 3:**
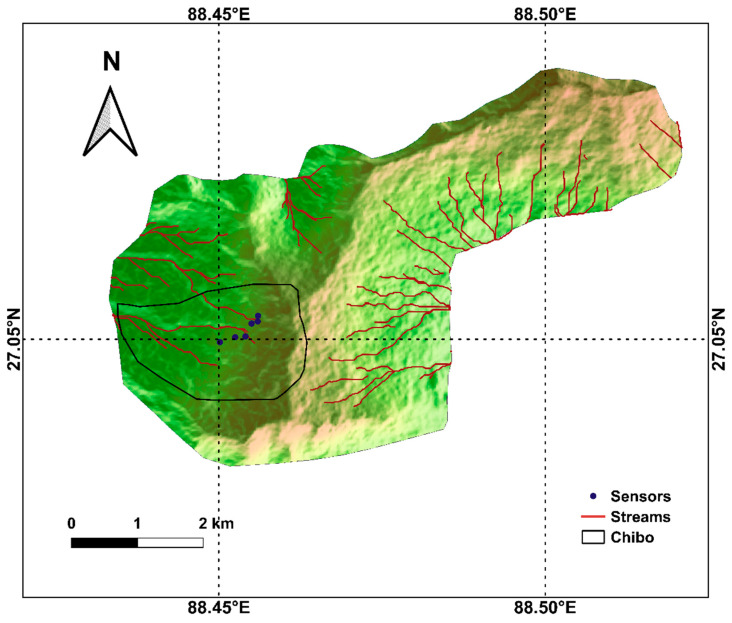
Drainage map of the region with the location of sensors.

The history of erosion and displacement near the two jhoras justifies the selection of sites for installing the sensors. The effect of rainfall and the flow of jhoras on displacement were monitored continuously for three monsoons from 2017 to 2019. Sensors 1, 2, and 3 are located near Pyarieni jhora, and the remaining three are near OC jhora. The location of sensors, along with the drainage and slope map of Kalimpong, is presented in Figure 3.

Sensor 2 is located in the right bank of Pyarieni jhora, and sensor 3 is on its left bank. Sensor 4 is placed on the left bank of OC jhora, and sensor 5 is also located nearby at the center of the Chibo area. The data logger is also located near sensor 6, which is placed near the OC jhora.

## 3. Field Monitoring

**Figure 4:**
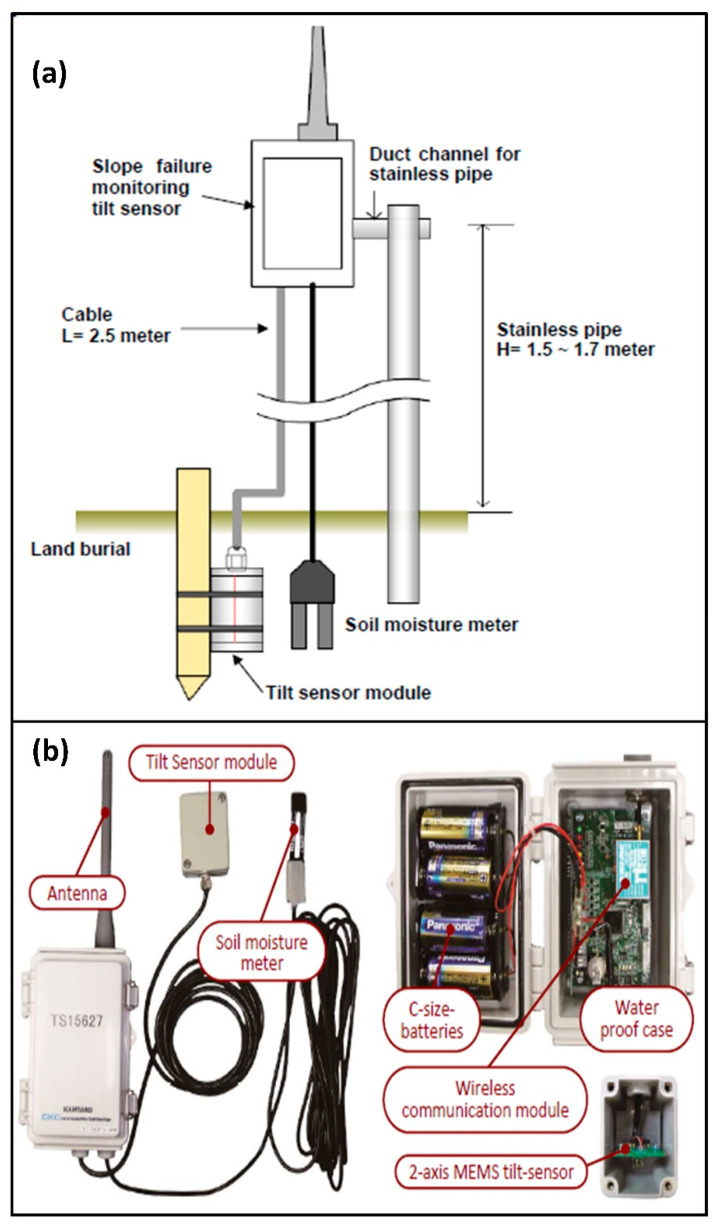
Details of tilt sensor: (**a**) schematic arrangement of the sensor set up and (**b**) components of a tilt sensor unit.

The monitoring system used in this study consists of six units. Each unit is equipped with a micro-electro-mechanical system (MEMS) that can measure the tilt angle of the module embedded within the soil [43]. A MEMS is a small integrated system with electrical and mechanical components. A volumetric water content sensor is also embedded in each unit, which measures the moisture content of the soil. The schematic arrangement of each unit and the components of the sensors are shown in Figure 4. The tilt sensors are placed with their abscissa parallel to the slope and ordinate perpendicular to the slope. The resolutions of sensors are mentioned in Table 1. The tilt sensor record tilting angle with an accuracy of 0.017° and the precision of volumetric moisture content is ±3%. The precision of measurement of sensors is subject to environmental conditions. The tilt sensors have a sensitivity of 4 V/g, which provides the output in digital voltage readings. Volumetric moisture content sensors have a response time of 10 ms, and the output is obtained as voltage value, similar to the tilt sensors. The voltage readings are then converted to tilting angles and volumetric moisture contents using conversion equations provided by the manufacturer. Power supply to the sensors is provided by four alkaline batteries of C size, which work in the field for more than a year. The low-cost sensor unit is easier to install and use than conventional extensometers, inclinometers, etc. The low cost is achieved using micro-electromechanical systems with integrated circuits, which are much smaller in dimension than conventional field monitoring techniques. It also uses dry cells, which are cheaper than the solar batteries. Energy consumption is reduced by letting the sensor sleep for a duration of 10 min after sending a signal wirelessly to the data logger. This will considerably increase the life of batteries in field [42] and thereby reduce the cost. The battery voltage is also transmitted in real time, allowing replacement before it is drained out. It can be installed at shallow depths, unlike the inclinometers, which requires deep boreholes for installation. In the case of extensometers, expertise is required to identify the location of installation, and it is often difficult for common people to identify the same without the support of an expert. In case of slope monitoring, where point data is received and many sensors are required to cover a region, cost-effective methods can only be used to foresee the failure part effectively. Apart from the advantages of being lightweight and easy to install, while comparing the performance of tiltmeters and extensometers, it was found that tiltmeters respond to the soil mass movements more quickly and hence can be used as a more reliable tool for early warning facilitation [42].

The volumetric water content sensors were placed at a depth of 30 cm in the slope. The moisture content is calculated indirectly by measuring the dielectric constant of the soil. The moisture content is measured at the point of contact of the sensor only whereas the tilt sensor measures the behavior of the soil around it.

A steel rod is placed with its bottom at 1 m below the surface. A wireless transmission kit is attached to the rod at its top, and the sensor is kept within a box embedded in soil. The bottom end is stable, and the top can undergo relative motion. For thin unstable layers, the depth of failing mass will be shorter. The average shear deformation of the surface layer is measured by the sensor as tilt angles. From the onset, the amount of displacement can be calculated, and the rate at which warnings are to be issued can be calculated from the observations.

The communication between the sensors and the web browser is enabled by machine to machine connection using IoT. The data collected from six different sensors can be used to provide landslide early warning. Radio communication is used to transfer the data from sensors wirelessly to a data logger. A wireless communication module is installed in each sensor unit for this purpose (Figure 4b). The communication range of each wireless communication module is 600 m, so the data logger is placed within this distance from each sensor. The data from all six sensors are gathered by the data logger, which is then transmitted to a data server via the internet. The data logger is a receiver that collects, saves, and transmits the data via the internet. It can receive data from 10 sensor units, and in this study, six sensor units are used. The system can be configured by specifying an offset time to avoid interferences due to multiple sensors. Each sensor is identified using a unique ID, and when the distance between data logger and sensor is more than 600 m, relays can be used in between. In such a case, separate IDs are used for relays as well. In this study, all sensors are located within 600 m from the data logger, so no relays are used. The IoT system connects the sensor units to the data logger and the internet database, enabling real-time monitoring of field data. This data can be used as an integral part of the LEWS along with the rainfall thresholds.

### Tilting Rate and Slope Failure

Xie et al. (2019) [23] conducted laboratory and field-scale experiments to predict possible slope failures by analyzing the tilting behavior of MEMS sensors. Artificial rainfall was used to trigger slope failure along a slip surface, which was pre-defined. An equation was proposed by the study in the form of Equation (1).

(1) $$\frac{dt}{\left|d\theta \right|}=\frac{-t}{B}+\frac{t_{f}}{B}$$

which can also be expressed as

(2) $$loglog\;\left(t_{f}-t\right)=-log\left(\frac{\left|d\theta \right|}{dt}\right)+B$$

where, $\frac{dt}{\left|d\theta \right|}$ is the inverse of tilting rates expressed in min/° and *t* is the time. *B* is the coefficient derived from the linear relationship between time and reciprocal tilting rate. At time *t_f_*, which is the time of slope failure, the reciprocal of tilting rate is assumed to be 0 min/°.

**Figure 5:**
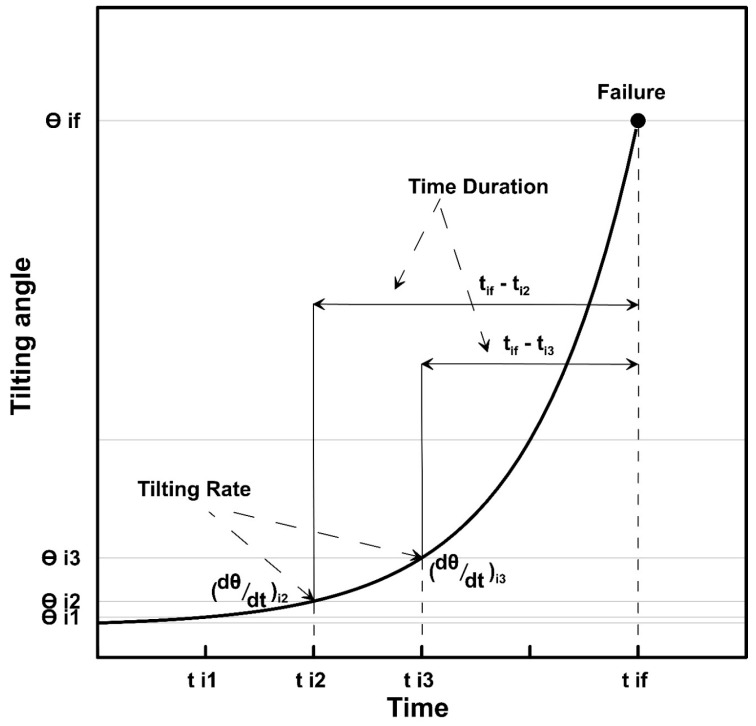
Definition of tilting rate and time duration (modified after Xie et al. (2019) [23]).

This equation will be used to evaluate the time duration within a span at a specific tilting rate before the failure. The graphical representation of the relationship between tilting rate and time duration is shown in Figure 5.

Hence, tilting rates can be considered an effective tool to predict the possible failure time. The direction of the tilt angles can also predict the possible failure plane, as the sensors placed above the failure plane tilts backward with the sliding slope, while the sensors reaching the slip surface are expected to tilt forward in the failure process. Tilt sensors placed at the lower part of the sliding masses can be used to detect the initiation of slope failure [23]. MEMS sensors are conventionally used to foresee the rapid shallow failures using this concept, but in this study, the displacements are found to be slow movements.

## 4. Data Used

**Figure 6:**
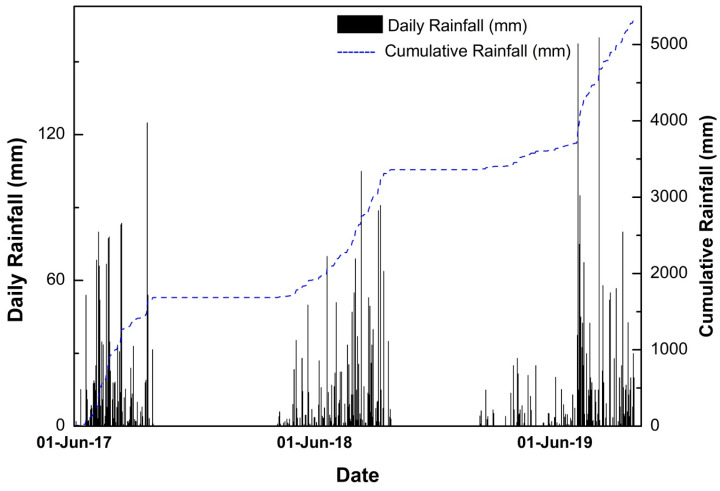
Time history of hourly and cumulative rainfall for the study period.

The slopes were monitored during three monsoons (1 July 2017 to 30 September 2019). This is beneficial in analyzing the effect of rainfall variation on the stability of slopes. The tilting angles of the sensors were found to vary during the monsoon seasons. It was observed that the rates are in good agreement with the precipitation measured in the study area. The rainfall data used for the analysis was collected from the rain gauge maintained by Save The Hills at Tirpai, Kalimpong [47]. A total of 5338.2 mm rainfall was observed in the region during the study period. The time history of daily and cumulative rainfall during study period is depicted in Figure 6. The monsoon season (June–September) contributed 90.78% of the total rainfall.

**Figure 7:**
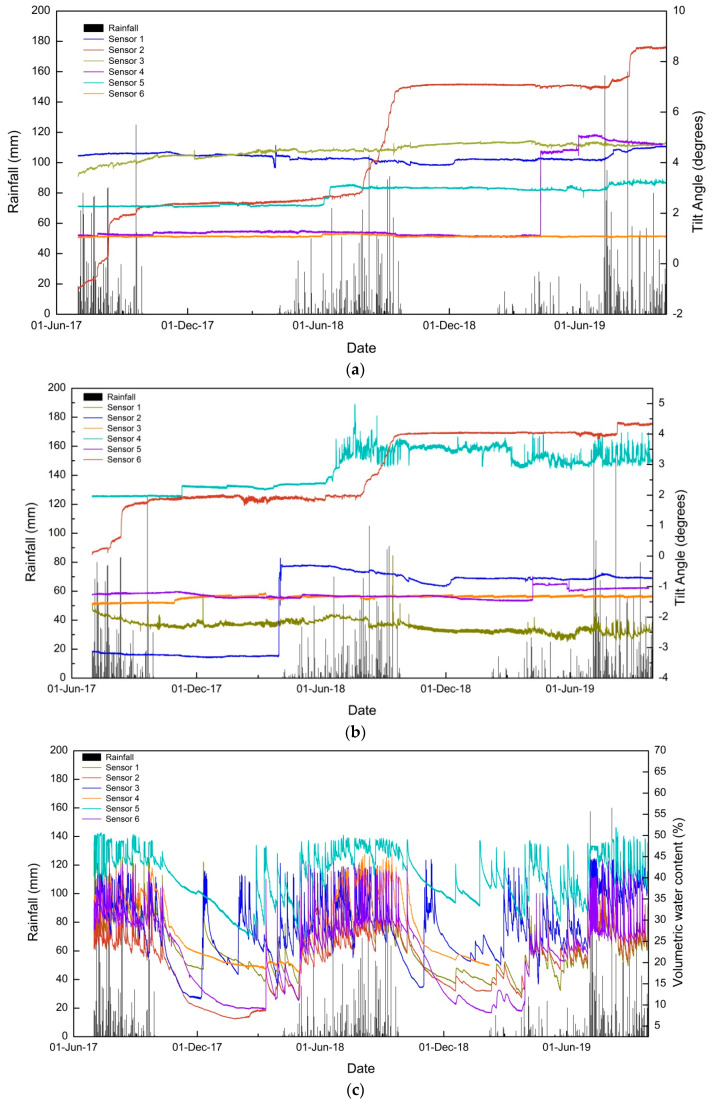
Data collected from six tilt sensors and volumetric water content sensors (2017–2019): (**a**) tilting angle in the direction parallel to the slope, (**b**) tilting angle in the direction perpendicular to the slope, and (**c**) volumetric moisture content.

From Figure 6, it can be inferred that the 2019 monsoon contributed the highest amount of rainfall, and according to India Meteorological Department (IMD), the amount of rainfall was 110% of the Long Period Average (LPA) rainfall [48] received in the country. The data from the tilt sensors (tilting angles in parallel and perpendicular directions) and the volumetric water content sensors are plotted in Figure 7a–c.

## 5. Results and Discussion

The detailed analysis of the data shows that sensor 2 showed the maximum variation in the 2017 and 2018 monsoons. In 2017, both sensor 3 and sensor 2 showed variations in tilting angle. These sensors are placed nearby, at the right and left of Pyarieni jhora, respectively. Sensor 4 and sensor 6 did not show any variations in tilting angles and are located at relatively stable areas. The field observations after each monsoon are also in accordance with the observed tilt angles. It can also be inferred that the tilting angle and observed displacements are in accordance with the rainfall received in the region. Rainfall induced scouring and erosion in the jhoras results in the slow subsidence of the upper hilly areas as well. Some sudden variations were observed in the tilting angles on days without rainfall and such readings are noted in sensor 1 during the 2017 monsoon and twice in sensor 4 during the 2019 monsoon, both in parallel and perpendicular directions. This can be attributed to possible external factors like human or animal interventions. The volumetric water content is the maximum near sensor 5. Another interesting observation is the fluctuating tilting angle of sensor 5 observed in 2018 and 2019. The higher concentration of moisture content results in unstable soil, which in turn leads to the oscillating behavior of tilting angle in a perpendicular direction. No such major displacements are observed near the sensor, and due to this, the observations from sensor 5 are discarded from the analysis. The detailed discussion regarding the variations of tilting angle for each monsoon season is as follows.

### 5.1. Monsoon 2017

**Figure 8:**
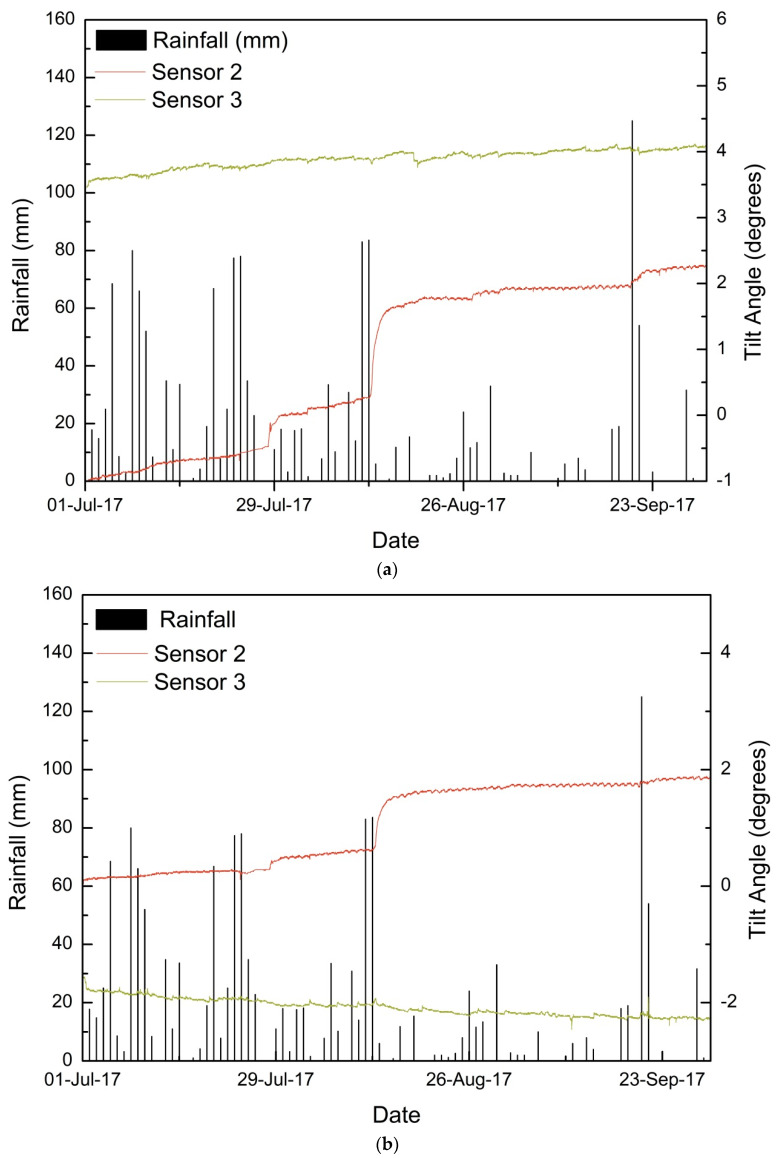

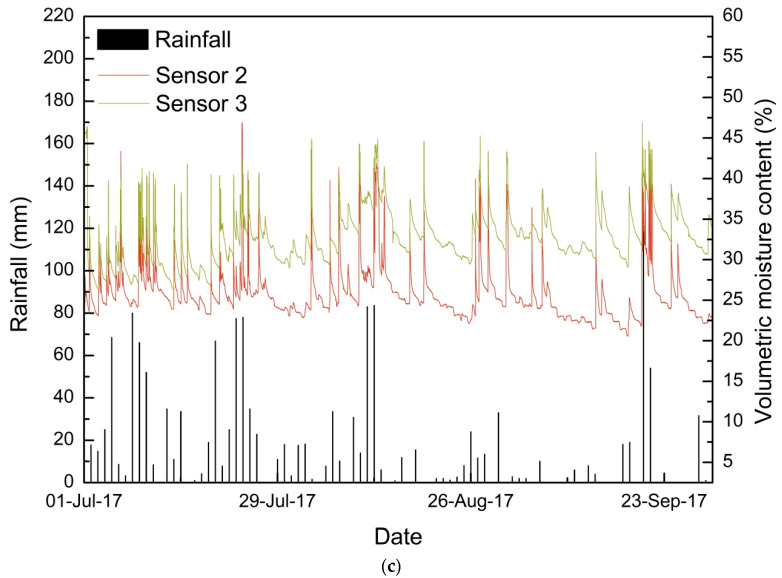
Detailed time history for monsoon 2017 of sensors 2 and 3: (**a**) tilting angle in the direction parallel to the slope, (**b**) tilting angle in a direction perpendicular to the slope, and (**c**) volumetric moisture content.

During the 2017 monsoon, the tilting angles of sensors 3 and 2 showed a notable increase in the tilt angles. On 28 and 29 July 2017, a major shift was observed in tilt angle from −0.477° to 0.006°. The initial tilting rate was 0.0117°/h which later reduced to 0.005°/h in the direction parallel to slope. The tilt angle of sensor 3 was found to be increasing at a very slow rate. The rainfalls on the days of displacement were found to be less, with no rain on 28 July 2017 and 11 mm rainfall on 29 July 2017. On the preceding days, starting from 17 July 2017, 336 mm rainfall was recorded until 27 July 2017. The maximum daily intensity of rainfall observed during the event was 77.4 mm. The antecedent rainfall increased the moisture content, as observed in Figure 8c. A second displacement period was noted from 13–17 August 2017. During this period, the tilt angle of the sensor increased from 1.126° to 1.714°. The average tilting rate was 0.007°/h on the first three days and 0.001°/h on the last two days. The maximum tilting rates during the initial period of displacement were 0.018°/h and 0.017°/h in the parallel and perpendicular directions, respectively. No sudden slope failures occurred during either of the displacement periods. The effect was observed as sinking of roads near the location of the sensor. The cumulative rainfall observed during the sinking period was only 23.6 mm while the antecedent rainfall for three days, five days, and 10 days prior to displacement were 180.6 mm, 211.4 mm, and 264.5 mm, respectively. Maximum water content was observed near sensor 3 during the second displacement period as 44%. The moisture content near sensor 2 remained less than that near sensor 3 during the whole monsoon period, but the variations in moisture content near sensor two was higher than those near sensor 3.

**Figure 9:**
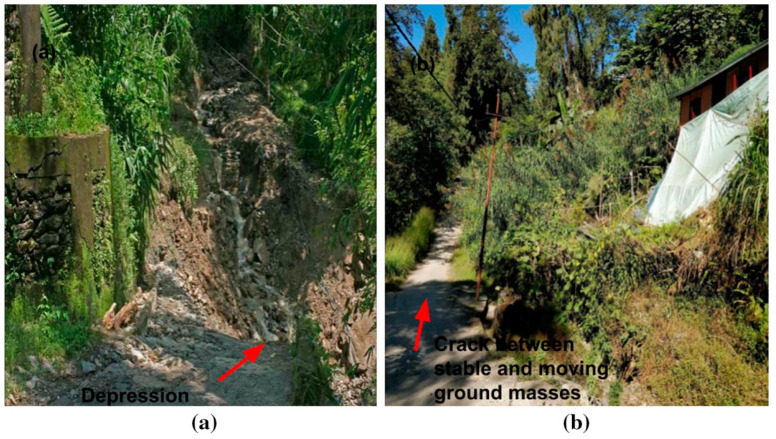

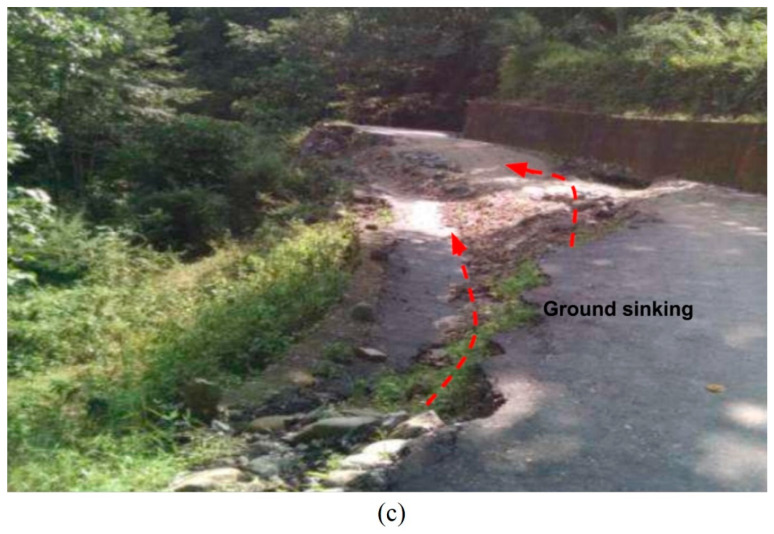
Displacements observed at Chibo after 2017 monsoon: (**a**) depression, (**b**) crack observed in ground, (**c**) ground sinking [22].

Figure 9 shows the displacements observed after the 2017 monsoon near the sensors. It was observed that near sensor 2, around 1 m subsidence occurred after the monsoon [22]. The regions near to sensor 2 (top left of Pyarieni jhora) are the most vulnerable among the six sensor locations. Also, the movement of tilt angles in the perpendicular direction of tilt sensors 2 and 3 are in opposite directions, which indicates a possible failure plane between the two. The hypothesis that subsidence and displacements are the results of scouring and erosion by the jhora in between the two sensors is strengthened by this observation.

### 5.2. Monsoon 2018

**Figure 10:**
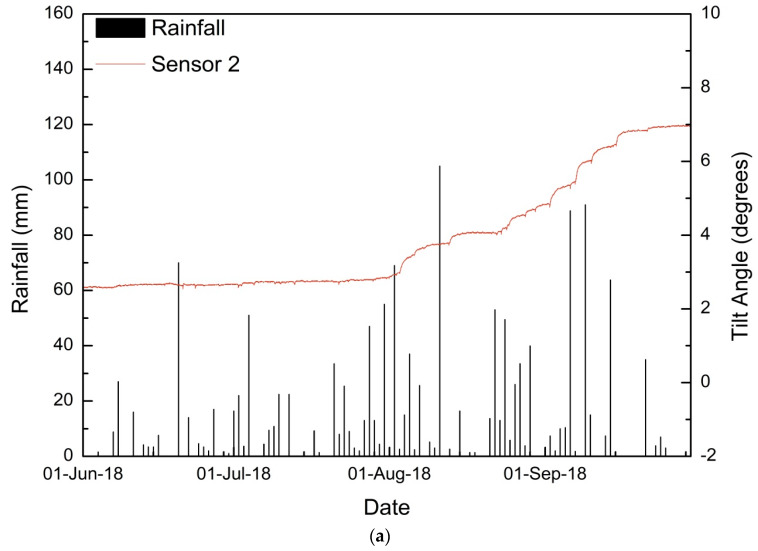

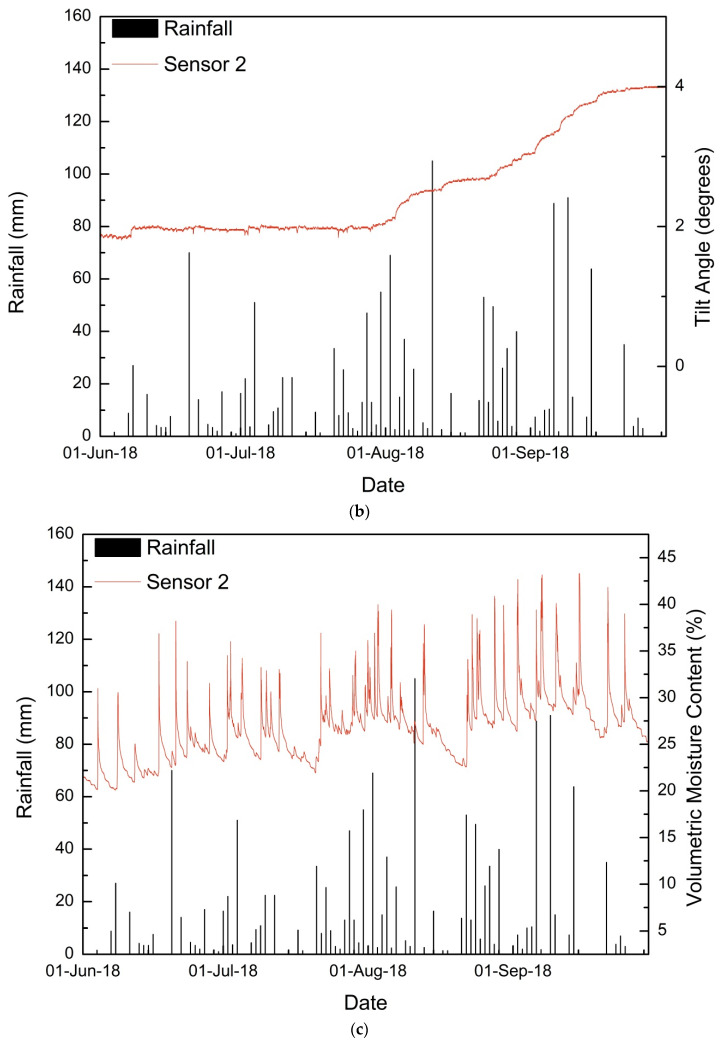
Detailed time history for monsoon 2018 of sensor 2: (**a**) tilting angle in the direction parallel to the slope, (**b**) tilting angle in the direction perpendicular to the slope, and (**c**) volumetric moisture content.

During the 2018 monsoon, sensor 2 showed the maximum variations in tilting angles. From 3 August to 15 September 2018, significant changes were observed in tilt angles, with a flat period from 14–24 August 2019. The same trend was observed in both parallel and perpendicular directions to the slope. The detailed time history of tilting angles and volumetric moisture content is shown in Figure 10.

For the first displacement period (3–8 August 2018), the total rainfall was 195.8 mm, with a maximum recorded precipitation of 105 mm on 11 August. The antecedent rainfall 3, 5, and 10 days prior to displacement were 124 mm, 141.4 mm, and 215.4 mm, respectively. The maximum tilting rate observed was 0.014°/h on 3 August 2018 in both parallel and perpendicular directions. The maximum moisture content was also recorded on 3 August as 39.97%. It can be inferred that the effect of continuous infiltration prior to the displacement period has more effect on the tilting rate as the maximum tilting was observed on a day with a mere 2.6 mm rainfall.

For the latter period (25 August 2019–15 September 2019), tilting was observed continuously for a period of 22 days at a slow rate. The maximum rate of tilting observed was 0.018°/h on 7 September 2018, followed by 0.0137°/h on 2 September 2018 and 0.01°/h on 15 September 2018. Similar to the displacements in the 2017 monsoon, the daily rainfall on 2, 7, and 15 September 2018 were 7.4 mm, 1.8 mm and 0 mm, respectively. The antecedent rainfall has resulted in higher water content on these three days as 40.89% on 2 September 2019, 43.16% on 7 September 2018, and 43.3% on 15 September 2018. This increase in water content has effectively reduced the strength and resulted in higher tilting rates.

**Figure 11:**
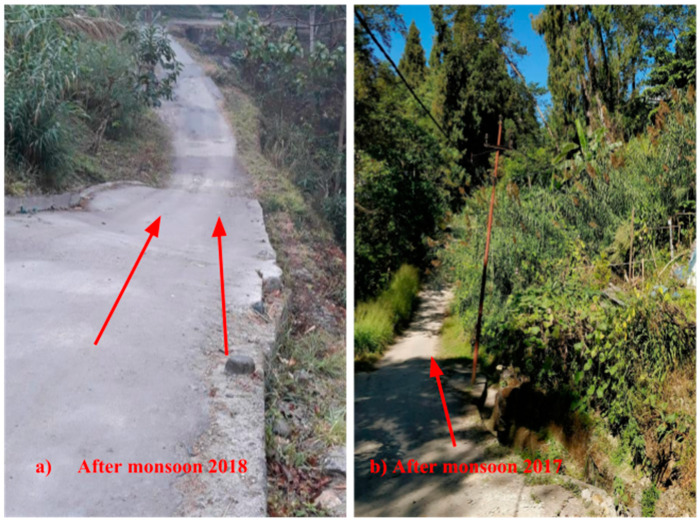
Displacements observed after 2018 and 2017 monsoons near sensor 2 (**a**) after the 2018 monsoon and (**b**) after the 2017 monsoon.

As shown in Figure 11, the roads near sensor 2 underwent substantial subsidence after the 2018 monsoon season. The other sensor locations remained relatively unaffected. The maximum tilting rate observed after two monsoons is 0.018°/h.

### 5.3. Monsoon 2019

**Figure 12:**
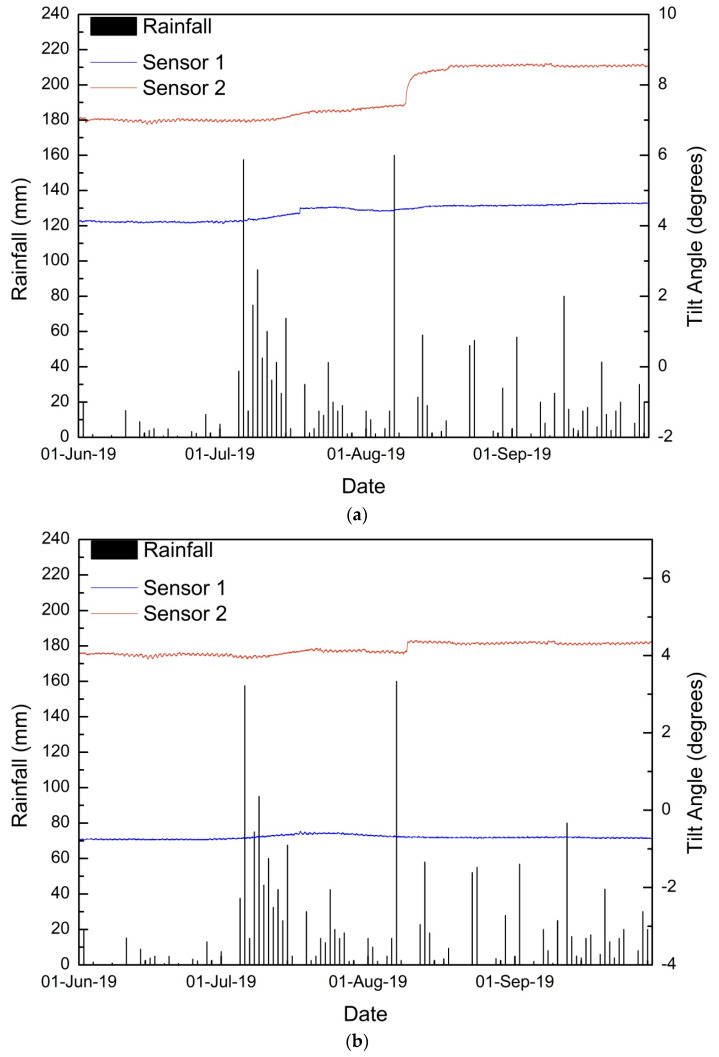

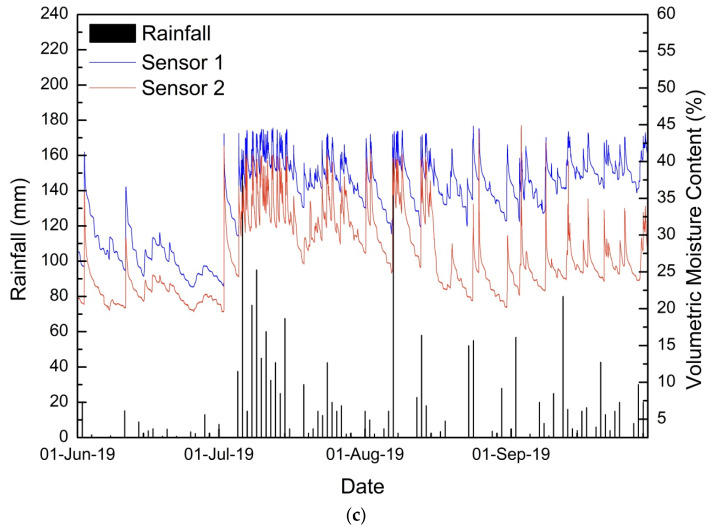
Detailed time history for the monsoon 2019 of sensors 1 and 2: (**a**) tilting angle in the direction parallel to the slope, (**b**) tilting angle in the direction perpendicular to the slope, and (**c**) volumetric moisture content.

Similar to the 2017 and 2018 monsoons, maximum tilting was observed in sensor 2 during the 2019 monsoon. Sensor 1 also recorded a minor increase in the tilting angle in the parallel direction to the slope. The detailed time history of tilting angles and moisture content are shown in Figure 12.

**Figure 13:**
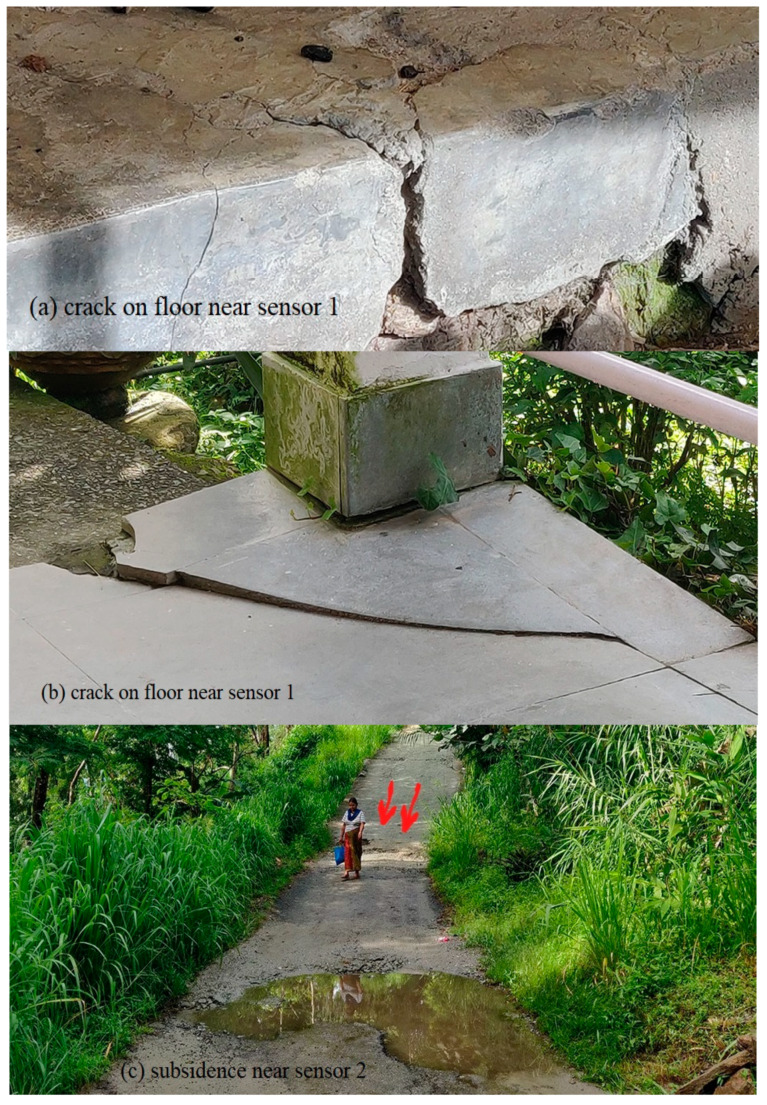
Displacements observed after the 2019 monsoon: (**a**) crack on floor near sensor 1, (**b**) crack on floor near sensor 1, (**c**) subsidence near sensor 2.

During the initial displacement period (12 July 2019–20 July2019), both sensors 1 and 2 tilt angles in a parallel direction increased at a very negligible rate. The maximum rate observed was 0.003°/h on 20 July 2019 in sensor 2. In sensor 1, a sudden shift in tilt angles was observed in 10 min from 4.362° to 4.502° on 17 August 2019 23:50. p.m., i.e., a tilting rate of 0.01395°/min, which is exceptionally high. The rate increased very slowly from 0.005°/h on 12 August 2019, and hence it was possible to monitor the sudden shift. Second displacement period was from 9–18 August 2019. A notable change in tilting angle was observed on 9 and 10 August 2019. In the parallel direction, a 0.0237°/h tilting rate was recorded on 9 August 2019, which reduced to 0.0096°/h on 10 August 2019. The tilting progressed at a minor rate until 20 August 2019. In the perpendicular direction, a sudden shift in tilt angle was observed on 9 August 2019 at a rate of 0.013°/h. No rainfall was recorded on 9 August 2019 in the study area and the antecedent rainfall on three days, five days, and 10 days before the peak tilting rate were 177.5 mm, 182.5 mm, and 209.5 mm, respectively. Figure 13 shows the displacements observed after monsoon 2019 near sensor 1 and sensor 2.

The displacement near sensor 2 was similar to those observed in 2017 and 2018, as subsidence in roads. In case of sensor 1, the observations are in accordance with the sudden shift observed in the tilt angle reading, i.e., cracks are observed on the floors of two houses nearby.

**Figure 14:**
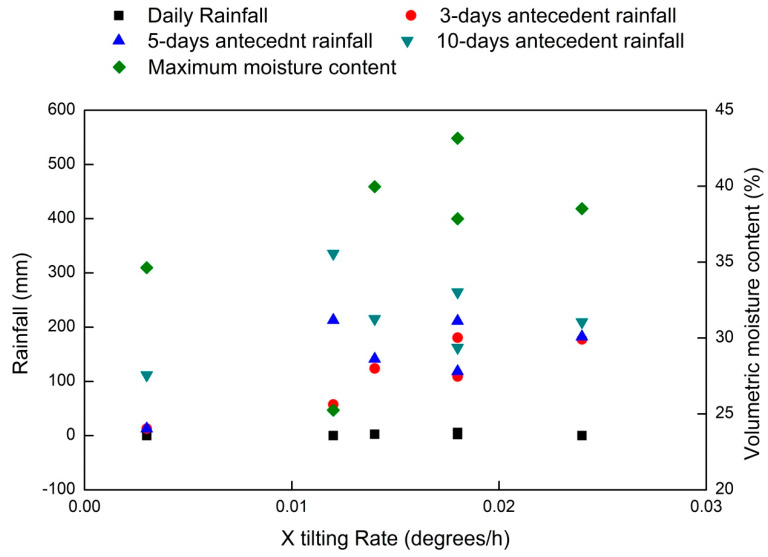
Tilting rate variation for sensor 2 for various displacement periods during study period with respect to rainfall and moisture conditions.

From Table 2, the variations in tilting rates with a daily rainfall, antecedent rainfall, and moisture content can be evaluated. The same has been plotted in Figure 14. From the summary, it is clear that antecedent rainfall plays a major role in ground displacement rather than daily rainfall values. Hence, the study points out the significance of using a statistical threshold that considers the effect of both short-term and long-term rainfall as the first line of early warning [49]. Such thresholds can also be conceptually modified by incorporating moisture content [8,14,50] and tilt angle values. The total rainfall received in the study area during the 2018 monsoon period was the least when compared to the other two monsoons. This is evident from the lesser value of antecedent rainfall observed in two displacement periods of 2018. However, continuous precipitation has created the same effect and has increased the tilting rates. All displacements have happened at tilting rates greater than 0.01°/h. The displacements observed were not rapid in nature, except the cracks observed near sensor 1 on 17 August 2019. Hence, the sensors prove effective in detecting very slow ground movements and can be used by the officials to be alert regarding the increasing tilting rates.

From Figure 14, it can be inferred that tilting rates in the X direction (parallel to slope) are higher than those in the Y direction (perpendicular to slope). The tilting rate is found to increase with the increase in volumetric moisture content as well. In all cases, the daily rainfall is clearly much less than the antecedent rainfall. The improper drainage system and water logging leads to an increase in moisture content, which increases the erosion rate. The erosion starts at the downhill area (near sensor 3) and is reflected as subsidence in the upper hilly areas (near sensor 2). The location near sensor 2 (Pyarieni jhora) is identified as the most critical area as it showed displacements in all three monsoon seasons.

## 6. Conclusions

The havoc associated with rainfall-induced landslides has severely disrupted the lives of the people in the Kalimpong area. Chibo is among the most affected areas within the town. The unstable slopes in this region were monitored regularly using six MEMS sensors for three monsoon seasons (2017–2019). The work is the first of its kind for the Darjeeling Himalayas, which are highly susceptible to landslides. It is necessary to evaluate the performance of this relatively new approach for the study area, as the first step of establishing an LEWS. The study presents the real-time monitoring data for three monsoon seasons in the Chibo area and explores the relationship between tilt rate and antecedent rainfall conditions. The monitoring data was compared with the field observations to evaluate the reliability of the approach.

Six tilt sensors and volumetric moisture content sensors were used to monitor the slopes near two mountain rivulets (jhoras) in Chibo. The data from the sensors are transferred in real time using wireless communications using IoT. The ground displacements observed after the monsoon season are in accordance with the tilt meter readings. The tilt sensors are found to be effective in monitoring very slow movements, and hence, it is possible to predict the failure time well in advance using real-time monitoring. It is also observed that variations in tilting are not always associated with peak rainfall intensities. Continuous precipitation and infiltration increase pore pressures and reduce the strength of the soil. Also, due to improper drainage, water channels are formed, which increases the erosion rate. The tilting rate is therefore associated with antecedent rainfall rather than the rainfall on the day of the maximum tilting rate. The study clearly points out to the significance of considering both long-term and short-term rainfall for developing rainfall thresholds for the region. This accounts for the higher number of false alarms reported for the conventional empirical thresholds defined for the study area [8,49]. In 2018, the rainfall received was the lowest when compared to the 2017 and 2019 monsoons. Still, the tilting rates remained comparable in all the three monsoons, and it can be inferred that, apart from rainfall conditions, local site conditions should also be considered when developing an effective early warning system. Some abrupt changes are observed in the tilt sensors when there is neither rainfall nor ground movements. Such changes can be due to animal/human interference. From an operational point of view, such sensors should therefore be monitored in real time by authorities to avoid false alarms.

## Figures and Tables

**Table 1:** Specifications of tilt sensor and volumetric moisture content sensor.

Sensor	Resolution
Tilt sensor	0.003°
Volumetric moisture content sensor	0.002 m^3^/m^3^

**Table 2:** Comparison of tilting rates with rainfall conditions at sensor 2.

Season	Daily Rainfall (mm)	3-Day Antecedent (mm)	5-Day Antecedent (mm)	10-Day Antecedent (mm)	Maximum Moisture Content (%)	X Tilting Rate (°/h)	Y Tilting Rate (°/h)
2017-1	0	57.6	213	335.8	25.25	0.012	0.012
2017-2	6	180.6	211.4	264.5	37.85	0.018	0.017
2018-1	2.6	124	141.4	215.4	39.97	0.014	0.014
2018-2	1.8	109.2	118.6	162.3	43.16	0.018	0.018
2019-1	0	12.8	12.8	111.6	34.63	0.003	0.005
2019-2	0	177.5	182.5	209.5	38.56	0.024	0.014
